# Exploring the relationship between gut microbiota and breast diseases using Mendelian randomization analysis

**DOI:** 10.3389/fmed.2024.1450298

**Published:** 2024-11-26

**Authors:** Xin Wang, Haoyu Gao, Yiyao Zeng, Jie Chen

**Affiliations:** ^1^Department of General Surgery, West China Hospital, Sichuan University, Chengdu, China; ^2^Breast Disease Center, West China Hospital, Sichuan University, Chengdu, China; ^3^Division of Cardiovascular Surgery, Department of General Surgery, West China Hospital, Sichuan University, Chengdu, China; ^4^Department of Cardiology, Dushu Lake Hospital Affiliated to Soochow University, Medical Center of Soochow University, Suzhou Dushu Lake Hospital, Suzhou, Jiangsu, China

**Keywords:** gut microbiota, breast cancer, breast diseases, Mendelian randomization, single nucleotide polymorphisms (SNPs)

## Abstract

**Background:**

Growing evidence suggests a relationship between gut microbiota composition and breast diseases, although the precise nature of this association remains uncertain. To investigate the causal relationship between gut microbiota and breast diseases, we utilized two-way Mendelian randomization (MR) analysis.

**Methods:**

Four common diseases were included as outcomes: breast cancer, breast cysts, inflammatory disorders of the breast, and infections of the breast associated with childbirth, along with their subtypes. Genetic data on gut microbiota were extracted from genome-wide association studies (GWAS). The primary approach used to investigate the association between these genetic factors and gut microbiota was the inverse-variance-weighted (IVW) method with random-effects types. Sensitivity analyses, such as Cochran’s Q test, the MR-Egger intercept test, and leave-one-out analysis, were conducted to ensure the stability and reliability of the MR findings.

**Results:**

We discovered plausible causal links between 20 microbial categories and the breast diseases, with a significance level of *p* < 0.05. Notably, *Family.Rikenellaceae* (*p*: 0.0013) maintained a significant inverse relationship with overall breast cancer (BC), after the Bonferroni correction. In the reverse MR analysis, interactions were observed between *Genus.Adlercreutzia* and estrogen receptor-positive cancer. In addition, *Genus.Sellimonas*, *Family.Rikenellaceae*, and *Genus.Paraprevotella* were associated with ER+ and overall breast cancer, whereas *Genus.Dorea* was linked to both estrogen receptor-negative and overall breast cancer. *Family.Prevotellaceae* was the only category correlated with inflammatory breast disorders. Moreover, *Genus Eubacteriumruminantiumgroup*, *Genus.Lactococcus*, and *Family.Alcaligenaceae* were associated with breast cysts, while *Genus.Anaerofilum*, *Genus.Butyricimonas*, *Order.Coriobacteriales*, *Order.Pasteurellales*, and *Order.Verrucomicrobiales* showed significant associations with infections of the breast associated with childbirth. No evidence of heterogeneity or horizontal pleiotropy was found.

**Conclusion:**

Our Mendelian randomization analysis confirmed a causal relationship between gut microbiota and breast diseases. Early stool tests may be a viable method for screening diseases to identify people at higher risk of breast diseases.

## Introduction

The community of microbes in the gastrointestinal (GI) ecosystem is known as gut microbiota (1). Humans are considered to have a symbiotic relationship with the gut microbiome. The gut microbiome has a complex composition and includes a wide variety of microorganisms, including more than 10^14^ bacteria, several archaea, eukaryotes, and viruses, all of which play a critical role in regulating human health and disease (2). Once an individual’s gut microbiome is in a state of metabolic disorder, resulting in composition imbalance and dysfunction, various diseases can occur (3, 4). Studies have shown that the gut microbiome is linked to various diseases, including diabetes (5), cancers (6), obesity (7), cardiovascular diseases (8), and immune diseases (9).

Current evidence suggests that there is a potential association between gut microbiota and breast diseases (10, 11). Growing evidence suggests a strong connection between gut microbiota and breast cancer (BC) (12, 13), which is the second most prevalent cancer worldwide and the most frequently diagnosed malignancy in women (14). Recent studies have also shown that intestinal bacteria may be transferred to the mammary gland through the entero-mammary pathway, in which immune cells transport intestinal bacteria to secondary lymph nodes and then to the mammary gland through blood or lymphatic circulation (15, 16). Some studies have reported that individuals with BC exhibit higher microbial diversity compared to healthy individuals (17) however, other studies have found lower microbial diversity in postmenopausal BC patients (18). Currently, some case–control studies rely on sequencing methods of specific regions within the bacterial *16S rRNA* gene to investigate the link between intestinal microbes and breast cancer. These studies have identified some possible pathogenic microbiota and suggested that this may be related to a reduced metabolic capacity of the microbiota and a weakened immune system (12, 19). Despite these findings, the composition of the microbiome community that causes cancer has not been determined. It is important to note that the available evidence, based on observational studies, is not strong enough to draw firm conclusions about the potential causal relationship between gut microbiota and cancer risk. However, there are few studies on benign breast lesions, such as fibroadenoma, mastitis, and breast cysts or abscesses.

Mendelian randomization (MR), an efficient technique for estimating exposure factors and outcomes (20, 21), has been employed to investigate the potential causal relationship between gut microbiota and various breast diseases. Recently, one study used MR analysis to explore the associations between 211 bacterial categories (encompassing 9 phyla, 20 orders, 16 classes, 36 families, and 131 genera) and BC^22^. Nonetheless, the potential causative relationship between various other gut microbiota categories and breast cancer remains uncertain, and the causal relationship between gut microbiota and other breast diseases has not been firmly established. We conducted a two-way MR investigation using genome-wide association studies (GWAS) data to probe the possible causal link between gut microbiota and a range of breast diseases, including breast cancer [both estrogen receptor-positive (ER+) and estrogen receptor-negative (ER-) breast cancer], breast cysts, inflammatory disorders of the breast, and infections of the breast associated with childbirth. We aimed to provide a theoretical basis for studying the causes of breast diseases in order to prevent the occurrence of breast cancer and other related diseases.

## Methods

### Data source

The GWAS data for BC were sourced from the Breast Cancer Association Consortium (BCAC), which comprised 1,22,977 cases and 1,05,974 controls, all of whom were of European descent (22). Regarding the primary subtypes of breast cancer, the data included information on 69,501 ER-positive (ER+) cases and 21,468 ER-negative (ER-) cases. Summary statistics from the GWAS study included breast cysts (4,61,145 cases and 4,62,933 controls), inflammatory disorders of the breast (757 cases and 1,15,030 controls), and infections of the breast associated with childbirth (456 cases and 1,19,115 controls).

The summarized statistics for the gut microbiota used in this investigation were extracted from the most recent GWAS meta-analysis, which included 18,340 participants from 24 cohorts and served as the exposure data (23). In summary, this study involved collaboration with experts in *16S rRNA* gene sequencing and incorporated genotyping information from over nine countries. In this study, association analyses were performed by consideringvariables such as age, technical factors, gender, and genetic principal components. As the study relied on publicly available aggregated data, there was no need for additional ethical approval or consent to participate.

### Selection of instrumental variables

This dataset includes a comprehensive collection of 211 gut microbial categories, which was categorized into five hierarchical levels—family, phylum, order, class, and genus. Among these categories, 15 were unidentified at the family or genus level and, as a result, were omitted, resulting in a total of 196 microbial categories for the Mendelian randomization analysis. Instrumental variables (IVs) with a *p*-value of less than 10^−5^ were selected because of the limited number of available single nucleotide polymorphisms (SNPs). To obtain the IVs from independent loci, we used the “TwoSampleMR” software package to set the linkage disequilibrium (LD) threshold with R^2^ < 0.001 and kb = 10,000. Subsequently, essential data, such as the effective allele and effective size (comprising *β* value, standard error, and *p*-value) of each SNP, were extracted for the computation of the F-statistic to assess the potential bias from weak instrumental variables (IVs). An F-statistic value exceeding 10 is considered adequate to mitigate any bias arising from weak IVs. When no expose-related SNPs were present in the outcome data, we conducted a follow-up analysis by finding and selecting suitable proxy SNPs (r^2^ > 0.8). In addition, the SNPs with palindromic structures were automatically excluded during the analysis. Finally, we conducted additional queries for these SNPs in the PhenoScanner database,^1^ excluding those linked to alternative potential confounders. We excluded 22 SNPs related to gender, educational attainment, smoking, body mass index, age at menarche, alcohol intake frequency, family history of cancer, and personal history of cancer.

### Statistical analysis

In our MR analysis, we assessed the association between gut microbiota and breast diseases using multiple statistical techniques, including the inverse-variance weighted (IVW) method with random-effects types, the MR-Egger test, and the MR-pleiotropy residual sum and outlier (MR-PRESSO) test. The IVW model is the primary analytical method for testing causality by performing a meta-analysis of each Wald ratio from valid SNPs included. This approach yields the most accurate effect estimates and serves as the primary analysis in nearly all MR investigations (24). In contrast, the MR-Egger analysis can still be effective even when all SNPS are invalid, which is evaluated as horizontal pleiotropy. The slope of the MR-Egger analysis indicates the relationship between gut microbiota and breast diseases when the intercept term is not statistically significant or zero. Cochran’s Q test is performed to assess the diversity among selected SNPs, and heterogeneity is indicated when the value is below 0.05.Furthermore, the MR-PRESSO test is used to conduct a comprehensive assessment for heterogeneity in order to identify potential anomalies within the SNP dataset. Following the identification and removal of these potential outliers, a corrected association result is obtained. To ensure a more stringent assessment of causality, we employed the Bonferroni correction to establish multiple testing significance thresholds across various taxonomic levels. These thresholds were determined based on the number of bacterial categories within each classification level, resulting in significance levels of (0.05/9) for phyla, (0.05/16) for classes, (0.05/20) for orders, (0.05/32) for families, and (0.05/119) for genera. A *p*-value that meets the threshold for nominal significance (less than 0.05) was considered to indicate a potential causal effect at the nominal level (25). To avoid horizontal pleiotropy caused by a single SNP, a leave-one-out analysis was performed. The analysis was conducted using the “TwoSampleMR” and “MR-PRESSO” packages within the R program (version 4.2.2). The statistical code is provided in Supplementary Table 3.

### Reverse analysis

**Figure 1:**
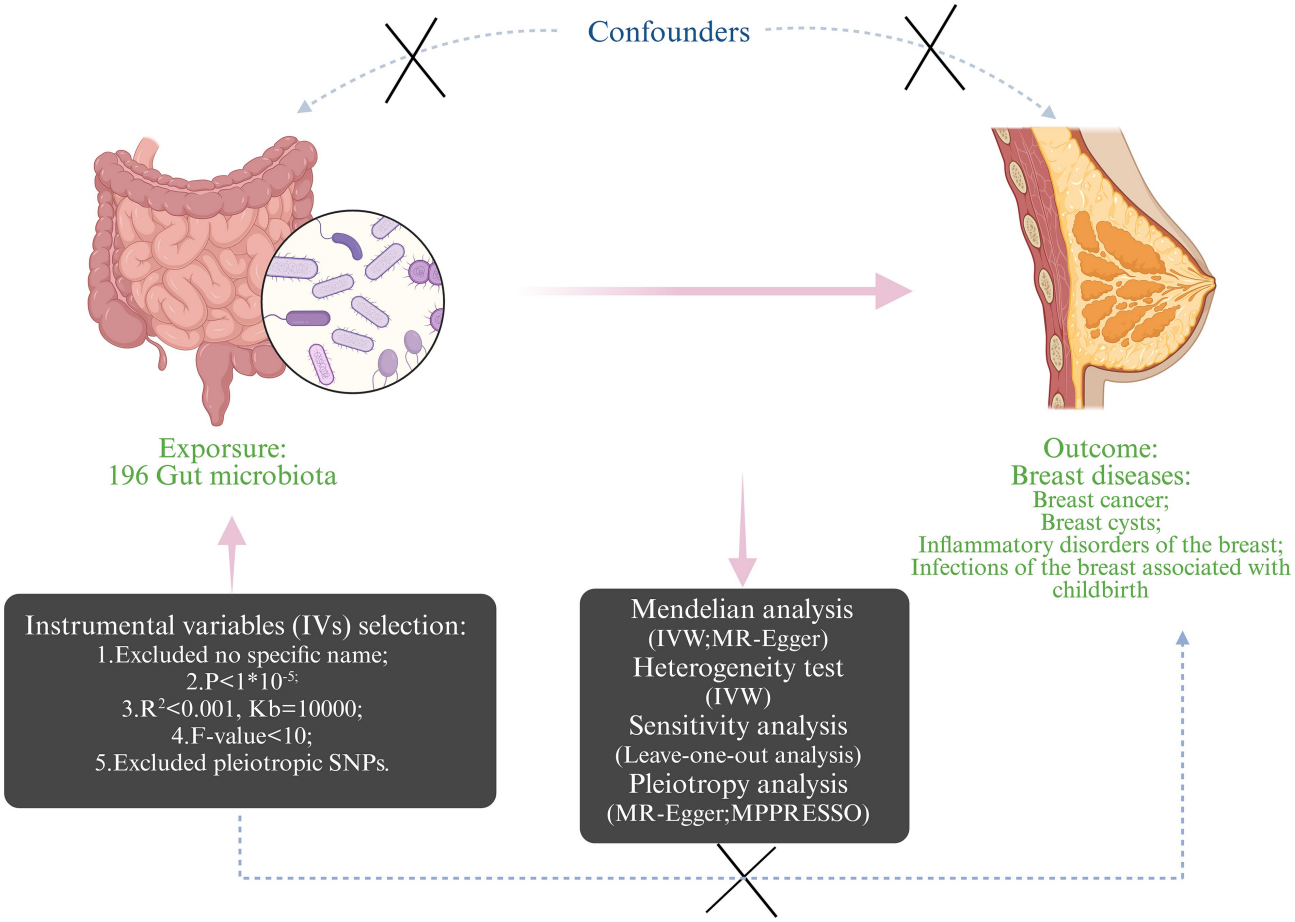
The design and workflow of the present Mendelian randomization study (Created with BioRender.com).

To explore potential bidirectional causality and assess reverse causation, reverse MR analyses were conducted, treating the breast diseases as the exposure and gut microbiota from the previous positive result as the outcomes. These reverse MR analyses utilized the same genome-wide association study (GWAS) datasets mentioned earlier. As for the MR analysis, a STROBE-MR checklist was completed and is summarized in Supplementary Table 1 (21). Figure 1 shows the specific MR study design.

## Results

### Overview of the MR analysis

In total, 196 microbial categories were included in the MR analysis. Following a rigorous instrumental selection step, the number of the SNPs linked to the bacterial categories varied between 10 and 122 (Table 1). In addition, the F-statistics were all above 10, indicating that the study did not rely on weak instrumental variables. The IVW method identified 20 microbial categories linked to the breast diseases (Table 1). Scatter plots illustrating the associations between these microbial categories and the breast diseases can be found in Supplementary Figures S1–S5.

**Table 1:** MR estimates from each method of assessing the causal effect between gut microbiota and breast diseases.

Exposure	Outcome	SNP (n)	Methods	Beta	SE	*p* val	OR(95% CI)
*Genus.Sellimonas*	Overall breast cancer	10	IVW	0.0466	0.0181	0.0098	1.0478(1.0112–1.0855)
			MR-Egger	0.0821	0.1131	0.4886	1.0856(0.8697–1.3550)
*Genus.Dorea*	Overall breast cancer	12	IVW	−0.1398	0.0454	0.0190	0.8695(0.7955–0.9505)
			MR-Egger	−0.1061	0.1337	0.9449	0.8993(0.6920–1.1688)
*Genus.Paraprevotella*	Overall breast cancer	82	IVW	−0.0253	0.0126	0.0446	0.9750(0.9512–0.9994)
			MR-Egger	−0.0802	0.0450	0.0783	0.9229(0.8450–1.0080)
*Family.Rikenellaceae*	Overall breast cancer	21	IVW	−0.0049	0.0436	0.0013	0.9951(0.9136–0.9939)
			MR-Egger	−0.1796	0.1477	0.2387	0.8356(0.6256–1.1162)
*Family.Ruminococcaceae*	Overall breast cancer	11	IVW	0.0115	0.0414	0.0071	1.0116(0.9327–1.0971)
			MR-Egger	0.2336	0.0870	0.0250	1.2631(1.0651–1.4980)
*Family.Streptococcaceae*	Overall breast cancer	15	IVW	−0.1042	0.0458	0.0229	0.9010(0.8237–0.9857)
			MR-Egger	−0.2498	0.1804	0.1894	0.7790(0.5470–1.1094)
*Phylum.Bacteroidetes*	Overall breast cancer	115	IVW	0.0506	0.0226	0.0252	1.0519(1.0063–1.0995)
			MR-Egger	−0.0012	0.0633	0.9855	0.9988(0.8823–1.1307)
*Genus.Sellimonas*	ER (+)	10	IVW	0.0681	0.0226	0.0026	1.0705(1.0241–1.1190)
			MR-Egger	0.0038	0.1420	0.9791	1.0038(0.7599–1.3259)
*Genus.Adlercreutzia*	ER (+)	22	IVW	0.0214	0.0265	0.0419	1.0216(0.9699–1.0761)
			MR-Egger	0.2432	0.0833	0.0085	1.2753(1.0832–1.5015)
*Genus.CandidatusSoleaferrea*	ER (+)	15	IVW	0.0527	0.0264	0.0459	1.0541(1.001–1.1101)
			MR-Egger	0.0348	0.1130	0.7620	1.0354(0.8297–1.2921)
*Genus.Paraprevotella*	ER (+)	82	IVW	0.0316	0.0156	0.0435	1.0321(1.001–1.0642)
			MR-Egger	−0.0679	0.0562	0.2304	0.9344(0.8369–1.0432)
*Family.Rikenellaceae*	ER (+)	21	IVW	−0.0882	0.0435	0.0428	0.9156(0.8408–0.9971)
			MR-Egger	0.0030	0.1457	0.9839	1.0030(0.7538–1.3345)
*Order.Bifidobacteriales*	ER (+)	115	IVW	−0.0333	0.0167	0.0451	0.9672(0.9361–0.9994)
			MR-Egger	0.0253	0.0463	0.5854	1.0256(0.9366–1.1231)
*Genus.Dorea*	ER (−)	12	IVW	−0.1597	0.0663	0.0188	0.8524(0.7485–0.9707)
			MR-Egger	−0.1638	0.1919	0.4134	0.8489(0.5828–1.2365)
*Genus.Eubacteriumruminantiumgroup*	Breast cyst	10	IVW	−0.0012	0.0006	0.0300	0.9988(0.9976–0.9999)
			MR-Egger	−0.0133	0.0068	0.0858	0.9868(0.9737–1.0000)
*Genus.Lactococcus*	Breast cyst	48	IVW	−0.0004	0.0002	0.0309	0.9996(0.9992–0.9999)
			MR-Egger	0.0309	−0.0002	0.0019	1.0314(1.0310–1.0318)
*Family.Alcaligenaceae*	Breast cyst	10	IVW	0.0018	0.0008	0.0220	1.0018(1.0002–1.0034)
			MR-Egger	0.0059	0.0085	0.5076	1.0059(0.9893–1.0228)
*Family.Prevotellaceae*	Inflammatory disorders of breast	18	IVW	−0.5393	0.2288	0.0184	0.5832(0.3724–0.9132)
			MR-Egger	−0.4732	0.7591	0.5418	0.6230(0.1407–2.7584)
*Genus.Anaerofilum*	Infections of breast associated with childbirth	12	IVW	0.4509	0.2106	0.0323	1.5697(1.0389–2.3719)
			MR-Egger	1.4389	0.9702	0.1688	4.2161(0.6296–28.2330)
*Genus.Anaerotruncus*	Infections of breast associated with childbirth	16	IVW	0.7718	0.3327	0.0203	2.1637(1.1272–4.1533)
			MR-Egger	−0.3339	1.011	0.7461	0.7161(0.0987–5.1948)
*Genus.Butyricimonas*	Infections of breast associated with childbirth	17	IVW	−0.5189	0.2625	0.0481	0.5952(0.3558–0.9956)
			MR-Egger	0.5495	1.0358	0.6035	1.7324(0.2775–13.1928)
*Order.Coriobacteriales*	Infections of breast associated with childbirth	122	IVW	−0.2809	0.1273	0.0272	0.7551(0.5884–0.9691)
			MR-Egger	−0.5987	0.3467	0.0867	0.5495(0.2785–1.0842)
*Order.Pasteurellales*	Infections of breast associated with childbirth	100	IVW	−0.2603	0.1048	0.0272	0.7708(0.6277–0.9466)
			MR-Egger	−0.3480	0.3079	0.2611	0.7061(0.3862–1.2911)
*Order.Verrucomicrobiales*	Infections of breast associated with childbirth	111	IVW	−0.2942	0.1167	0.0117	0.7451(0.5928–0.9366)
			MR-Egger	−0.0530	0.3473	0.8789	0.9484(0.4801–1.8733)

### Overall breast cancer

**Figure 2:**
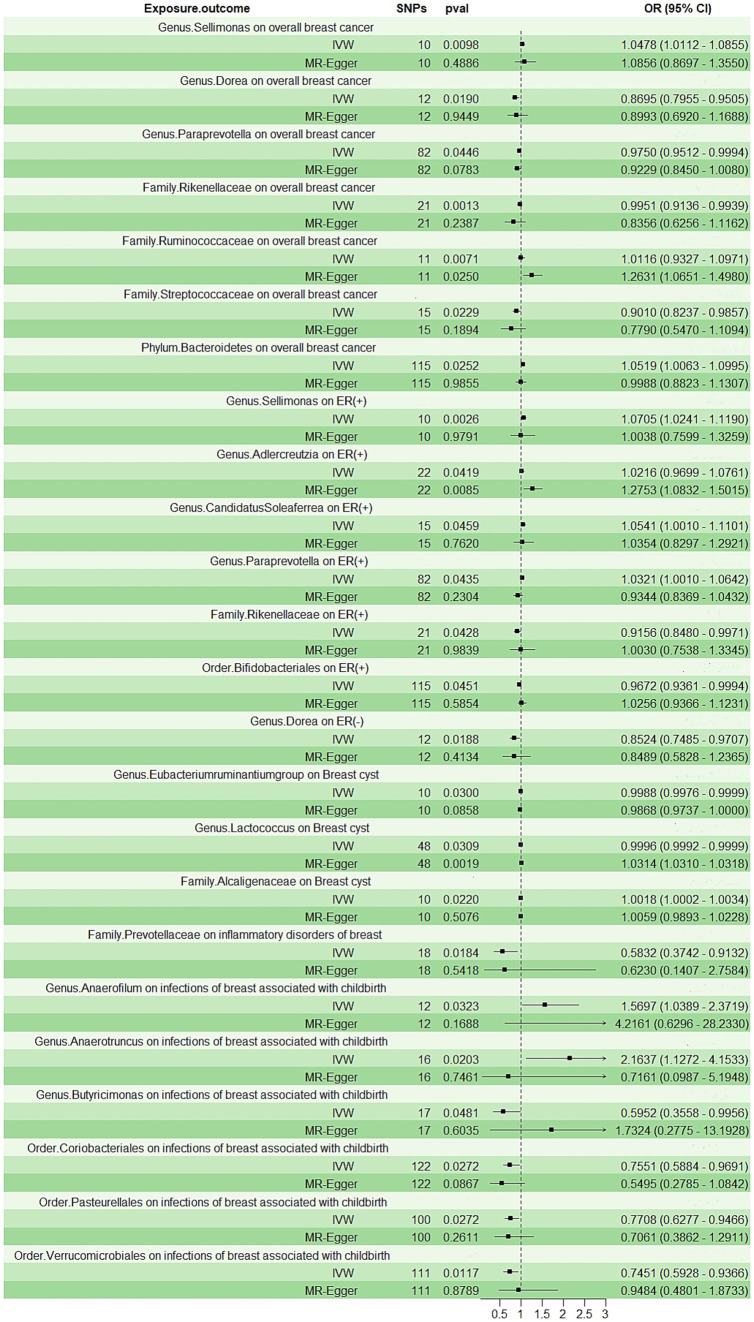
The forest plot shows the odds ratios and 95% confidence intervals (CIs) for the effect of the gut microbiota on the breast diseases.

The IVW analysis indicated that *Genus.Sellimonas* (Odds Ratio: 1.0478, 95% confidence interval (CI): 1.0112–1.0855), *Family.Ruminococcaceae* (Odds Ratio: 1.0116, 95% CI: 0.9327–1.0971), and *Phylum.Bacteroidetes* (Odds Ratio: 1.0591, 95% CI: 1.0063–1.0995) showed an association with an elevated risk of overall breast cancer (*p* < 0.05), whereas *Genus.Dorea* (Odds Ratio: 0.8695, 95% CI: 0.7955–0.9505), *Genus.Paraprevotella* (Odds Ratio: 0.9750, 95% CI: 0.9512–0.9994), *Family.Rikenellaceae* (Odds Ratio: 0.9951, 95% CI: 0.9136–0.9939), and *Family.Streptococcaceae* (Odds Ratio: 0.9010, 95% CI: 0.8237–0.9857) showed an association with a decreased overall breast cancer risk (*p* < 0.05; Figure 2). Nevertheless, only *Family.Ruminococcaceae* maintained consistent results in the MR-Egger analysis (Supplementary Figure S1).

### ER (+) and ER (−)

According to the IVW method (Figure 2; Supplementary Figure S2), *Genus.Sellimonas* (Odds Ratio: 1.0705, 95% CI: 1.0241–1.1190), *Genus.Adlercreutzia* (Odds Ratio: 1.0116, 95% CI: 0.9699–1.0761), *Genus.CandidatusSoleaferrea* (Odds Ratio: 1.0541, 95% CI: 1.001–1.1101), and *Genus.Paraprevotella* (Odds Ratio: 1.0321, 95% CI: 1.001–1.0642) exhibited associations with an elevated risk of ER-positive (+) breast cancer (*p* < 0.05). However, *Order.Bifidobacteriales* (Odds Ratio: 0.9672, 95% CI: 0.9361–0.9994) and *Genus.Paraprevotella* (Odds Ratio: 0.8524, 95% CI: 0.7485–0.9707) were related to a reduced risk of ER (+) and ER (−) breast cancer, respectively (*p* < 0.05; Figure 2). Only *Genus.Adlercreutzia* maintained consistent results in the MR-Egger analysis (Supplementary Figure S2).

### Breast cysts and inflammatory disorders of the breast

*Genus.Eubacteriumruminantiumgroup* (Odds Ratio: 0.9988, 95% CI: 0.9976–0.9999) and *Genus.Lactococcus* (Odds Ratio: 0.9996, 95% CI: 0.9992–0.9999) were negatively correlated with breast cyst risk in the IVW approach (*p* < 0.05; Figure 2). Only *Family.Alcaligenaceae* (Odds Ratio: 1.0018, 95% CI: 1.0002–1.0034) was observed to be positively associated with breast cyst risk in the IVW analysis (*p* < 0.05; Figure 2). *Family.Prevotellaceae* (Odds Ratio: 0.5832, 95% CI: 0.3724–0.9132) was associated with a reduced risk of inflammatory disorders of the breast, but we did not find any positive associations (Figure 2; Supplementary Figure S4). Only *Genus.Lactococcus* maintained consistent results in the MR-Egger analysis (Supplementary Figure S3).

### Infections of the breast associated with childbirth

Two categories, namely *Genus.Anaerofilum* (Odds Ratio: 1.5697, 95% CI: 1.0389–2.3719) and *Genus.Anaerotruncus* (Odds Ratio: 2.1637, 95% CI: 1.1272–4.1533), were positively associated with the risk of infections of the breast associated with childbirth (*p* < 0.05; Figure 2; Supplementary Figure S5). Conversely, we observed that inverse associations with *Genus.Butyricimonas* (Odds Ratio: 0.5952, 95% CI: 0.3558–0.9956), *Order.Coriobacteriales* (Odds Ratio: 0.7551, 95% CI: 0.5884–0.9691), *Order.Pasteurellales* (Odds Ratio: 0.7788, 95% CI: 0.6277–0.9466), and *Order.Verrucomicrobiales* (Odds Ratio: 0.7451, 95% CI: 0.5928–0.9366) exhibited a negative correlation with the risk of infections of the breast associated with childbirth (*p* < 0.05; Figure 2; Supplementary Figure S5).

### The Bonferroni correction, reverse analysis, and sensitivity analysis

Notably, when the Bonferroni correction was employed to adjust for multiple comparisons across various classification levels, the results confirmed that *Family.Rikenellaceae* maintained a significant inverse association with overall breast cancer (Odds Ratio: 0.9951, 95% CI: 0.9136–0.9939, *p* = 0.0013). Only one gut microbiota was identified after the reverse MR analysis, which indicated that ER+ breast cancer was associated with *Genus.Adlercreutzia* (Odds ratio:1.0682, 95%CI:1.0121–1.1273, *p*: 0.0166). The detailed reverse MR analysis is summarized in Supplementary Table 2. Both Cochran’s Q test and the MR-PRESSO test indicated the absence of detected heterogeneity (*p* > 0.05; Table 2). The *p*-values of the intercept terms in the MR-Egger regression analysis all exceeded 0.05, indicating that there was no potential horizontal pleiotropy. The result from the leave-one-out analysis indicated that no single SNP played a decisive role in the causal inference between the gut microbiota and breast diseases (Supplementary Figures S6–S10).

**Table 2:** Cochran Q, Horizontal pleiotropy and MR-PRESSO test of MR analysis.

Exposure	Outcome	SNP (n)	Cochran’s Q			Horizontal pleiotropy			MR-PRESSO test	
			Q	Q_df	Q_pval	Egger intercept	se	*p*-value	RSSobs	*p*-value
*Genus.Sellimonas*	Overall breast cancer	10	8.8412	9	0.4521	−0.0052	0.0162	0.7584	8.7834	0.876
*Genus.Dorea*	Overall breast cancer	12	17.0930	11	0.1051	−0.0071	0.0093	0.4586	8.1020	0.321
*Genus.Paraprevotella*	Overall breast cancer	82	125.9835	81	0.0010	0.0052	0.0041	0.2075	19.3720	0.198
*Family.Rikenellaceae*	Overall breast cancer	21	23.6091	20	0.2599	0.0028	0.0100	0.7804	11.8721	0.282
*Family.Ruminococcaceae*	Overall breast cancer	11	12.7825	10	0.2361	−0.0103	0.0066	0.1518	9.2820	0.182
*Family.Streptococcaceae*	Overall breast cancer	15	8.0174	14	0.8884	0.0115	0.0138	0.4191	9.1719	0.883
*Phylum.Bacteroidetes*	Overall breast cancer	115	163.2078	114	0.0017	0.0033	0.0037	0.3835	46.8033	0.214
*Genus.Sellimonas*	ER (+)	10	9.9254	9	0.3566	0.00933	0.0203	0.6587	7.8920	0.219
*Genus.Adlercreutzia*	ER (+)	22	33.3964	20	0.3050	−0.0027	0.0010	0.1187	11.903	0.321
*Genus.CandidatusSoleaferrea*	ER (+)	15	32.6075	20	0.0372	−0.0065	0.0099	0.5194	7.9800	0.192
*Genus.Paraprevotella*	ER (+)	82	135.5997	81	0.0001	0.0035	0.0051	0.5027	13.1995	0.772
*Family.Rikenellaceae*	ER (+)	21	16.6055	14	0.2778	0.00183	0.0112	0.8724	19.0212	0.297
*Order.Bifidobacteriales*	ER (+)	115	158.0474	114	0.0040	−0.0041	0.0030	0.1770	13.1864	0.762
*Genus.Dorea*	ER (−)	12	9.1103	11	0.6117	0.0003	0.0134	0.9822	6.9220	0.563
*Order.Desulfovibrionales*	ER (−)	97	101.4091	90	0.3331	0.0050	0.0050	0.5611	101.4091	0.333
*Genus.Eubacteriumruminantiumgroup*	Breast cyst	10	7.9154	9	0.5427	−0.0002	0.0005	0.6426	8.5410	0.686
*Genus.Lactococcus*	Breast cyst	48	32.4253	47	0.9478	−1.8445e-05	0.0002	0.9227	19.2920	0.922
*Family.Alcaligenaceae*	Breast cyst	10	5.2512	7	0.6293	0.0005	0.0006	0.4249	4.8220	0.238
*Family.Prevotellaceae*	Inflammatory disorders of breast	18	19.2933	17	0.3120	−0.0050	0.0546	0.9282	6.2390	0.176
*Genus.Anaerofilum*	Infections of breast associated with childbirth	12	6.9779	11	0.8009	−0.1155	0.1107	0.3214	8.1570	0.818
*Genus.Anaerotruncus*	Infections of breast associated with childbirth	16	13.0931	15	0.5951	0.0779	0.0673	0.2662	15.0621	0.607
*Genus.Butyricimonas*.	Infections of breast associated with childbirth	17	13.9073	16	0.6056	−0.0891	0.0835	0.3031	15.6534	0.63
*Order.Coriobacteriales*	Infections of breast associated with childbirth	122	96.8199	121	0.9483	0.0221	0.0225	0.3263	98.4867	0.944
*Order.Pasteurellales*	Infections of breast associated with childbirth	100	93.5487	99	0.6330	0.0076	0.0251	0.7625	95.6948	0.627
*Order.Verrucomicrobiales*	Infections of breast associated with childbirth	111	123.2931	110	0.1822	−0.1932	0.0262	0.4625	125.4258	0.189

## Discussion

Our two-way MR study identified a total of 20 bacterial categories potentially associated with the risk of the breast diseases, including *Genus.Sellimonas*, *Genus.Dorea*, *Genus.Adlercreutzia*, *Genus.Paraprevotella*, *Genus.Anaerofilum*, *Genus.Anaerotruncus*, *Genus.Lactococcus*, *Genus.CandidatusSoleaferrea*, *Order.Verrucomicrobiales*, *Genus.Butyricimonas*, *Genus.Eubacteriumruminantiumgroup*, *Family.Rikenellaceae*, *Family.Alcaligenaceae*, *Family.Prevotellaceae*, *Family.Ruminococcaceae*, *Family.Streptococcaceae*, *Phylum.Bacteroidetes*, *Order.Bifidobacteriales*, *Order.Coriobacteriales*, and *Order.Pasteurellales*. One of these taxa, *Family.Rikenellaceae*, displayed robust potential causality, as determined by the Bonferroni correction. Our study is the first to use MR analysis to comprehensively reveal the potential causal relationship between the gut microbiota and breast diseases, including not only breast cancer but also other breast conditions.

Regarding overall breast cancer, our study showed that *Genus.Sellimonas*, *Family.Ruminococcaceae*, and *Phylum.Bacteroidetes* exhibited a potential causal link with an elevated risk of breast cancer, while *Genus.Dorea*, *Genus.Paraprevotella*, *Family.Rikenellaceae*, and *Family.Streptococcaceae* were associated with a reduced risk of breast cancer. Moreover, we found compelling evidence of causal associations between *Genus.Sellimonas*, *Genus.Adlercreutzia*, *Genus.CandidatusSoleaferrea*, *Order.Bifidobacteriales*, and *Genus.araprevotella* and both ER (+) and ER (−) breast cancer. Ruminococcaceae was found at higher levels, while *Dorea* was found at lower levels in BC patients, according to a case–control study by Goedert et al. (18) Intestinal dysbiosis is associated with higher levels of circulating estrogen in postmenopausal breast cancer. Microbial species of Clostridia, Ruminococcaceae, and *Escherichia* have been found to be associated with estrogen metabolism (26). In addition, by examining the levels of non-ovarian systemic estrogen, Flores et al. found a strong association between breast cancer and Ruminococcaceae in postmenopausal women (27). The results are consistent with the findings of our study. Bacteroidetes are the predominant phyla implicated in the metabolism of indigestible nutrients in the colon, including dietary fiber and polyphenols, which are associated with obesity (28). Furthermore, obesity increases postmenopausal breast cancer risk and mortality, and it also increases estrogen and inflammatory mediators that contribute to aggressive breast cancer (29). However, the relationship between obesity and breast cancer remains unclear. Studies have shown that Streptococcaceae levels are significantly higher in healthy human tissues compared to those in breast cancer patients (30). Certain research has revealed positive associations between the abundance of *Streptococcus* and the existence of *β*-glucuronidase, an enzyme that interferes with the binding of estrogen to other substances, thereby rendering it a biologically active hormone (31). This disruption of the estrogen group has been demonstrated to influence estrogen and its metabolites at both local and systemic levels, which in turn has been linked to the risk of BC (32). There have been limited studies on *Sellimonas*. It has been reported to be overrepresented in stool samples from patients with more aggressive tumors, suggesting that *Sellimonas* has a potential carcinogenic effect in human hosts (33). The underlying mechanism is worth exploring in the future.

An imbalance in sex hormones is recognized as a primary risk factor for BC. In addition, female sex hormone levels influence microbiota composition, although this relationship works both ways. A category of bacteria known as the estrogen group plays a role in the synthesis of beta-glucuronidase and influences the regulation of estrogen metabolism, distribution, and elimination. This category includes *Bifidobacterium*, *Edwardsiella*, *Collinsella*, *Alistipes*, *Clostridium*, and *Bacteroides* (34). As for *Paraprevotella*, studies have found that they were mostly beneficial bacteria (35). Previous studies have shown that, compared to malignant and benign breast diseases, breast stromal tissue has a higher fat content and a reduced fibrotic component. In addition, *Adlercreutzia* has been found to be positively correlated with the percentage of fibrosis by several orders of magnitude (36). The results of previous studies align with our study’s findings, which indicated that *Adlercreutzia* was the only significant gut bacterium identified in our reverse analysis. According to a previous study, the absolute numbers of *Bifidobacteriales* vary according to clinical stages, suggesting that the composition of gut microbiota could be linked to the onset of BC^19^. These bacteria are recognized as one of the “beneficial” bacterial members and have been observed to play an anti-tumor role, correlating with an increased anti-PD-L1 therapeutic response (37). Further exploration is needed.

Our investigation also indicated that *Family.Alcaligenaceae* exhibited a potential causal connection with an elevated risk of breast cysts, whereas *Genus.Eubacteriumruminantiumgroup* and *Genus.Lactococcus* were linked with a reduced risk of breast cysts. Zhijun Ma et al. found that *Lactococcus* was reduced in patients with benign breast lesions compared to healthy individuals, and it is generally considered a beneficial bacterium, which is consistent with our research findings (11). Several studies have demonstrated the role of *Lactococcus* in the regulation of specific immune processes in the context of tumor development. *Lactococcus* regulates cellular immunity by maintaining the cytotoxic activity of innate natural killer (NK) cells. It has been shown to enhance cellular immunity by activating key cells associated with tumor growth (38). Nonetheless, there is a lack of prior research examining the association between *Family.Alcaligenaceae*, *Genus.Eubacteriumruminantiumgroup*, and breast cyst. Further exploration is needed in this area of research.

We observed an inverse association between *Family.Prevotellaceae*, which is known as a butyrate producer, and inflammatory breast disorders. The mechanism of their interaction is not yet clear, but microorganisms can contribute to inflammation. The gut microbiota may function by stimulating chronic inflammation, modifying the equilibrium between host cell growth and apoptosis, and triggering unregulated innate and adaptive immune reactions. The study identified a plausible inflammatory biomarker, mucosecretory immunoglobulin A (IgA), which helps maintain the integrity of the mucosal barrier by recognizing and regulating the composition of gut microbiota. This, in turn, reduces its adaptability and mitigates the host’s innate immune response (39).

In addition, some gut bacteria, including *Genus.Anaerofilum* and *Genus.naerotruncus,* were found to be positively correlated with infections of the breast associated with childbirth, while *Genus.Butyricimonas*, *Order.Coriobacteriales*, *Order.Pasteurellales*, and *Order.Verrucomicrobiales* were negatively associated. Human breasts are not sterile and contain a diverse and unique bacterial community (40). To date, some findings have demonstrated that part of the breast tissue microbiota is derived from the gastrointestinal tract, the nipple–areolar area, and nipple–mouth contact through breastfeeding and/or sexual contact (41). Therefore, there may be some intestinal bacteria associated with infections of the breast. In addition, research findings have shown that alterations in *Anaeotruncus* affect the rate of breast cancer (42), while our research also highlights its impact on breast infections.

Recently, two related MR studies also found that *Genus.Sellimonas* predicted a higher risk of overall and ER (+) BC (33, 43). These findings are consistent with the results of our study. However, they also indicated that *Parabacteroides*, *Genus.Eubacteriumxylanophilumgroup*, and *Desulfovibrio* were risk factors for breast cancer, which were not included in our study results. Our research not only identified bacteria related to breast cancer that were not found in previous studies but also examined the relationship between other breast diseases and intestinal flora. This is the first study to analyze these associations using MR analysis. Our study extended the existing body of knowledge regarding the causal link between the gut microbiota and breast diseases, offering substantial evidence. The primary results of our study indicate that stool testing could serve as a viable approach for identifying individuals at elevated risk of breast diseases, further supporting the need for more frequent and thorough testing. Although the detected associations did not reach the level of significance required for the Bonferroni correction, the potential impacts of these gut microbiota should not be disregarded. These findings may instead indicate a bacterial composition with potential carcinogenic properties, which could aid in assessing disease risk and identifying specific gut microbiota candidates for future functional investigations, especially in the context of cancer and inflammatory diseases.

However, our study has some limitations. First, our analyses only included individuals of European descent, making it difficult to evaluate whether these results can be extrapolated to other racial groups. Second, although we used the largest GWAS dataset to extract the SNPs, only a small amount of variation could be explained by instrumental variables, resulting in limited statistical power. Third, even after employing the MR-Egger method, we could not completely rule out the misclassification of genetic polymorphisms. Fourth, the ultimate outcomes were derived through the rigorous Bonferroni correction, a method implemented to minimize the likelihood of false-positive findings. Fifth, for two-sample MR studies, there might have been over-identification and overestimation of the correlation between the exposure and SNPs (44). Finally, the gut microbiota might have been influenced by environmental or genetic factors, which limited the variance in the interpretation of genetic instruments.

## Conclusion

In summary, this Mendelian randomization study highlights the potential causal involvement of gut microbiota in the onset of breast diseases. Certain bacteria are considered prospective candidates for clinical intervention. Early stool tests may serve as a viable method for screening diseases to identify individuals at higher risk of breast diseases. Future research is necessary to further investigate the underlying mechanisms.

## Data Availability

The original contributions presented in the study are included in the article/Supplementary material, further inquiries can be directed to the corresponding author.

## Footnotes

^1^ http://www.phenoscanner.medschl.cam.ac.uk/
