# Supplementary material for: Exploring the relationship between gut microbiota and breast diseases using Mendelian randomization analysis
Source: Front Med (Lausanne). 2024 Nov 26;11:1450298. doi: 10.3389/fmed.2024.1450298 (PMC11654425; doi:10.3389/fmed.2024.1450298)
Supplement: Supplementary file 1 [file Table_1.DOCX]

Table 1. MR estimates from each method of assessing the causal effect between gut microbiota and breast diseases.

| **Exposure** | **Outcome** | **SNP (n)** | **Methods** | **Beta** | **SE** | **pval** | **OR (95% CI)** |
| --- | --- | --- | --- | --- | --- | --- | --- |
| Genus.Sellimonas | Overall breast cancer | 10 | IVW | 0.0466 | 0.0181 | 0.0098 | 1.0478(1.0112-1.0855) |
|  |  |  | MR-Egger | 0.0821 | 0.1131 | 0.4886 | 1.0856(0.8697-1.3550) |
| Genus.Dorea | Overall breast cancer | 12 | IVW | -0.1398 | 0.0454 | 0.0190 | 0.8695(0.7955-0.9505) |
|  |  |  | MR-Egger | -0.1061 | 0.1337 | 0.9449 | 0.8993(0.6920-1.1688) |
| Genus.Paraprevotella | Overall breast cancer | 82 | IVW | -0.0253 | 0.0126 | 0.0446 | 0.9750(0.9512-0.9994) |
|  |  |  | MR-Egger | -0.0802 | 0.0450 | 0.0783 | 0.9229(0.8450-1.0080) |
| Family.Rikenellaceae | Overall breast cancer | 21 | IVW | -0.0049 | 0.0436 | 0.0013 | 0.9951(0.9136-0.9939) |
|  |  |  | MR-Egger | -0.1796 | 0.1477 | 0.2387 | 0.8356(0.6256-1.1162) |
| Family.Ruminococcaceae | Overall breast cancer | 11 | IVW | 0.0115 | 0.0414 | 0.0071 | 1.0116(0.9327-1.0971) |
|  |  |  | MR-Egger | 0.2336 | 0.0870 | 0.0250 | 1.2631(1.0651-1.4980) |
| Family.Streptococcaceae | Overall breast cancer | 15 | IVW | -0.1042 | 0.0458 | 0.0229 | 0.9010(0.8237-0.9857) |
|  |  |  | MR-Egger | -0.2498 | 0.1804 | 0.1894 | 0.7790(0.5470-1.1094) |
| Phylum.Bacteroidetes | Overall breast cancer | 115 | IVW | 0.0506 | 0.0226 | 0.0252 | 1.0519(1.0063-1.0995) |
|  |  |  | MR-Egger | -0.0012 | 0.0633 | 0.9855 | 0.9988(0.8823-1.1307) |
| Genus.Sellimonas | ER (+) | 10 | IVW | 0.0681 | 0.0226 | 0.0026 | 1.0705(1.0241-1.1190) |
|  |  |  | MR-Egger | 0.0038 | 0.1420 | 0.9791 | 1.0038(0.7599-1.3259) |
| Genus.Adlercreutzia | ER (+) | 22 | IVW | 0.0214 | 0.0265 | 0.0419 | 1.0216(0.9699-1.0761) |
|  |  |  | MR-Egger | 0.2432 | 0.0833 | 0.0085 | 1.2753(1.0832-1.5015) |
| Genus.CandidatusSoleaferrea | ER (+) | 15 | IVW | 0.0527 | 0.0264 | 0.0459 | 1.0541(1.001-1.1101) |
|  |  |  | MR-Egger | 0.0348 | 0.1130 | 0.7620 | 1.0354(0.8297-1.2921) |
| Genus.Paraprevotella | ER (+) | 82 | IVW | 0.0316 | 0.0156 | 0.0435 | 1.0321(1.001-1.0642) |
|  |  |  | MR-Egger | -0.0679 | 0.0562 | 0.2304 | 0.9344(0.8369-1.0432) |
| Family.Rikenellaceae | ER (+) | 21 | IVW | -0.0882 | 0.0435 | 0.0428 | 0.9156(0.8408-0.9971) |
|  |  |  | MR-Egger | 0.0030 | 0.1457 | 0.9839 | 1.0030(0.7538-1.3345) |
| Order.Bifidobacteriales | ER (+) | 115 | IVW | -0.0333 | 0.0167 | 0.0451 | 0.9672(0.9361-0.9994) |
|  |  |  | MR-Egger | 0.0253 | 0.0463 | 0.5854 | 1.0256(0.9366-1.1231) |
| Genus.Dorea | ER (-) | 12 | IVW | -0.1597 | 0.0663 | 0.0188 | 0.8524(0.7485-0.9707) |
|  |  |  | MR-Egger | -0.1638 | 0.1919 | 0.4134 | 0.8489(0.5828-1.2365) |
| Genus.Eubacteriumruminantiumgroup | Breast cyst | 10 | IVW | -0.0012 | 0.0006 | 0.0300 | 0.9988(0.9976-0.9999) |
|  |  |  | MR-Egger | -0.0133 | 0.0068 | 0.0858 | 0.9868(0.9737-1.0000) |
| Genus.Lactococcus | Breast cyst | 48 | IVW | -0.0004 | 0.0002 | 0.0309 | 0.9996(0.9992-0.9999) |
|  |  |  | MR-Egger | 0.0309 | -0.0002 | 0.0019 | 1.0314(1.0310-1.0318) |
| Family.Alcaligenaceae | Breast cyst | 10 | IVW | 0.0018 | 0.0008 | 0.0220 | 1.0018(1.0002-1.0034) |
|  |  |  | MR-Egger | 0.0059 | 0.0085 | 0.5076 | 1.0059(0.9893-1.0228) |
| Family.Prevotellaceae | Inflammatory disorders of breast | 18 | IVW | -0.5393 | 0.2288 | 0.0184 | 0.5832(0.3724-0.9132) |
|  |  |  | MR-Egger | -0.4732 | 0.7591 | 0.5418 | 0.6230(0.1407-2.7584) |
| Genus.Anaerofilum | Infections of breast associated with childbirth | 12 | IVW | 0.4509 | 0.2106 | 0.0323 | 1.5697(1.0389-2.3719) |
|  |  |  | MR-Egger | 1.4389 | 0.9702 | 0.1688 | 4.2161(0.6296-28.2330) |
| Genus.Anaerotruncus | Infections of breast associated with childbirth | 16 | IVW | 0.7718 | 0.3327 | 0.0203 | 2.1637(1.1272-4.1533) |
|  |  |  | MR-Egger | -0.3339 | 1.011 | 0.7461 | 0.7161(0.0987-5.1948) |
| Genus.Butyricimonas | Infections of breast associated with childbirth | 17 | IVW | -0.5189 | 0.2625 | 0.0481 | 0.5952(0.3558-0.9956) |
|  |  |  | MR-Egger | 0.5495 | 1.0358 | 0.6035 | 1.7324(0.2775-13.1928) |
| Order.Coriobacteriales | Infections of breast associated with childbirth | 122 | IVW | -0.2809 | 0.1273 | 0.0272 | 0.7551(0.5884-0.9691) |
|  |  |  | MR-Egger | -0.5987 | 0.3467 | 0.0867 | 0.5495(0.2785-1.0842) |
| Order.Pasteurellales | Infections of breast associated with childbirth | 100 | IVW | -0.2603 | 0.1048 | 0.0272 | 0.7708(0.6277-0.9466) |
|  |  |  | MR-Egger | -0.3480 | 0.3079 | 0.2611 | 0.7061(0.3862-1.2911) |
| Order.Verrucomicrobiales | Infections of breast associated with childbirth | 111 | IVW | -0.2942 | 0.1167 | 0.0117 | 0.7451(0.5928-0.9366) |
|  |  |  | MR-Egger | -0.0530 | 0.3473 | 0.8789 | 0.9484(0.4801-1.8733) |

Table 2. Cochran Q, Horizontal pleiotropy and MR-PRESSO test of MR analysis.

| **Exposure** | **Outcome** | **SNP (n)** | **Cochran's Q** | | | **Horizontal pleiotropy** | | | **MR-PRESSO test** | |
| --- | --- | --- | --- | --- | --- | --- | --- | --- | --- | --- |
|  |  |  | **Q** | **Q_df** | **Q_pval** | **Egger intercept** | **se** | **p-value** | **RSSobs** | **p-value** |
| Genus.Sellimonas | Overall breast cancer | 10 | 8.8412 | 9 | 0.4521 | -0.0052 | 0.0162 | 0.7584 | 8.7834 | 0.876 |
| Genus.Dorea | Overall breast cancer | 12 | 17.0930 | 11 | 0.1051 | -0.0071 | 0.0093 | 0.4586 | 8.1020 | 0.321 |
| Genus.Paraprevotella | Overall breast cancer | 82 | 125.9835 | 81 | 0.0010 | 0.0052 | 0.0041 | 0.2075 | 19.3720 | 0.198 |
| Family.Rikenellaceae | Overall breast cancer | 21 | 23.6091 | 20 | 0.2599 | 0.0028 | 0.0100 | 0.7804 | 11.8721 | 0.282 |
| Family.Ruminococcaceae | Overall breast cancer | 11 | 12.7825 | 10 | 0.2361 | -0.0103 | 0.0066 | 0.1518 | 9.2820 | 0.182 |
| Family.Streptococcaceae | Overall breast cancer | 15 | 8.0174 | 14 | 0.8884 | 0.0115 | 0.0138 | 0.4191 | 9.1719 | 0.883 |
| Phylum.Bacteroidetes | Overall breast cancer | 115 | 163.2078 | 114 | 0.0017 | 0.0033 | 0.0037 | 0.3835 | 46.8033 | 0.214 |
| Genus.Sellimonas | ER (+) | 10 | 9.9254 | 9 | 0.3566 | 0.00933 | 0.0203 | 0.6587 | 7.8920 | 0.219 |
| Genus.Adlercreutzia | ER (+) | 22 | 33.3964 | 20 | 0.3050 | -0.0027 | 0.0010 | 0.1187 | 11.903 | 0.321 |
| Genus.CandidatusSoleaferrea | ER (+) | 15 | 32.6075 | 20 | 0.0372 | -0.0065 | 0.0099 | 0.5194 | 7.9800 | 0.192 |
| Genus.Paraprevotella | ER (+) | 82 | 135.5997 | 81 | 0.0001 | 0.0035 | 0.0051 | 0.5027 | 13.1995 | 0.772 |
| Family.Rikenellaceae | ER (+) | 21 | 16.6055 | 14 | 0.2778 | 0.00183 | 0.0112 | 0.8724 | 19.0212 | 0.297 |
| Order.Bifidobacteriales | ER (+) | 115 | 158.0474 | 114 | 0.0040 | -0.0041 | 0.0030 | 0.1770 | 13.1864 | 0.762 |
| Genus.Dorea | ER (-) | 12 | 9.1103 | 11 | 0.6117 | 0.0003 | 0.0134 | 0.9822 | 6.9220 | 0.563 |
| Order.Desulfovibrionales | ER (-) | 97 | 101.4091 | 90 | 0.3331 | 0.0050 | 0.0050 | 0.5611 | 101.4091 | 0.333 |
| Genus.Eubacteriumruminantiumgroup | Breast cyst | 10 | 7.9154 | 9 | 0.5427 | -0.0002 | 0.0005 | 0.6426 | 8.5410 | 0.686 |
| Genus.Lactococcus | Breast cyst | 48 | 32.4253 | 47 | 0.9478 | -1.8445e-05 | 0.0002 | 0.9227 | 19.2920 | 0.922 |
| Family.Alcaligenaceae | Breast cyst | 10 | 5.2512 | 7 | 0.6293 | 0.0005 | 0.0006 | 0.4249 | 4.8220 | 0.238 |
| Family.Prevotellaceae | Inflammatory disorders of breast | 18 | 19.2933 | 17 | 0.3120 | -0.0050 | 0.0546 | 0.9282 | 6.2390 | 0.176 |
| Genus.Anaerofilum | Infections of breast associated with childbirth | 12 | 6.9779 | 11 | 0.8009 | -0.1155 | 0.1107 | 0.3214 | 8.1570 | 0.818 |
| Genus.Anaerotruncus | Infections of breast associated with childbirth | 16 | 13.0931 | 15 | 0.5951 | 0.0779 | 0.0673 | 0.2662 | 15.0621 | 0.607 |
| Genus.Butyricimonas. | Infections of breast associated with childbirth | 17 | 13.9073 | 16 | 0.6056 | -0.0891 | 0.0835 | 0.3031 | 15.6534 | 0.63 |
| Order.Coriobacteriales | Infections of breast associated with childbirth | 122 | 96.8199 | 121 | 0.9483 | 0.0221 | 0.0225 | 0.3263 | 98.4867 | 0.944 |
| Order.Pasteurellales | Infections of breast associated with childbirth | 100 | 93.5487 | 99 | 0.6330 | 0.0076 | 0.0251 | 0.7625 | 95.6948 | 0.627 |
| Order.Verrucomicrobiales | Infections of breast associated with childbirth | 111 | 123.2931 | 110 | 0.1822 | -0.1932 | 0.0262 | 0.4625 | 125.4258 | 0.189 |
