# Supplementary material for: Exploring the relationship between gut microbiota and breast diseases using Mendelian randomization analysis
Source: Front Med (Lausanne). 2024 Nov 26;11:1450298. doi: 10.3389/fmed.2024.1450298 (PMC11654425; doi:10.3389/fmed.2024.1450298)
Supplement: Supplementary file 2 [file Table_2.DOCX]

Supplemental Table 2. The reverse mendelian randomization between breast diseases and gut microbiota.

| Exposure | Outcome | SNP (n) | Methods | Beta | SE | pval | OR (95% CI) |
| --- | --- | --- | --- | --- | --- | --- | --- |
| Overall breast cancer | Genus.Sellimonas | 75 | IVW | 0.0641 | 0.0441 | 0.1460 | 1.0662(0.9779-1.1624) |
| Overall breast cancer | Genus.Dorea | 75 | IVW | -0.0104 | 0.0187 | 0.5786 | 0.9897(0.9540-1.0266) |
| Overall breast cancer | Genus.Paraprevotella | 75 | IVW | -0.0159 | 0.0294 | 0.5893 | 0.9842(0.9291-1.0427) |
| Overall breast cancer | Family.Rikenellaceae | 75 | IVW | 0.0070 | 0.0207 | 0.7347 | 1.0071(0.9699-1.0489) |
| Overall breast cancer | Family.Ruminococcaceae | 75 | IVW | -0.0072 | 0.0187 | 0.7014 | 0.9929(0.9572-1.0299) |
| Overall breast cancer | Family.Streptococcaceae | 75 | IVW | -0.0354 | 0.0222 | 0.1101 | 0.9652(0.9242-1.0081) |
| Overall breast cancer | Phylum.Bacteroidetes | 75 | IVW | 0.0122 | 0.0196 | 0.5333 | 1.0123(0.9741-1.0519) |
| ER (+) | Genus.Sellimonas | 52 | IVW | 0.0603 | 0.0445 | 0.1756 | 1.0621(0.9734-1.1590) |
| ER (+) | Genus.Adlercreutzia | 52 | IVW | 0.0659 | 0.0275 | 0.0166 | 1.0682(1.0121-1.1273) |
| ER (+) | Genus.CandidatusSoleaferrea | 52 | IVW | 0.0256 | 0.0321 | 0.4243 | 1.0260(0.9634-1.0925) |
| ER (+) | Genus.Paraprevotella | 52 | IVW | -0.0039 | 0.0299 | 0.8965 | 0.9961(0.9394-1.0563) |
| ER (+) | Family.Rikenellaceae | 52 | IVW | 0.0029 | 0.0219 | 0.8956 | 1.0029(0.9607-1.0469) |
| ER (+) | Order.Bifidobacteriales | 52 | IVW | 0.0069 | 0.0235 | 0.7673 | 1.0070(0.9617-1.0544) |
| ER (-) | Genus.Dorea | 13 | IVW | 0.0443 | 0.0291 | 0.1280 | 1.0453(0.9874-1.1066) |
| ER (-) | Order.Desulfovibrionales | 13 | IVW | 0.0456 | 0.0308 | 0.1390 | 1.0466(0.9853-1.1118) |
| Breast cyst | Genus.Eubacteriumruminantiumgroup | 6 | IVW | 0.0467 | 0.0312 | 0.2390 | 1.0324(0.9767-1.0786) |
| Breast cyst | Genus.Lactococcus | 6 | IVW | 0.0012 | 0.0439 | 0.8762 | 1.0034(0.9678-1.0512) |
| Breast cyst | Family.Alcaligenaceae | 6 | IVW | 0.0078 | 0.0245 | 0.7879 | 1.0081(0.9717-1.0549) |
| Inflammatory disorders of breast | Family.Prevotellaceae | 6 | IVW | 0.0712 | 0.0557 | 0.1876 | 1.0723(0.9812-1.163) |
| Infections of breast associated with childbirth | Genus.Anaerofilum | 8 | IVW | 0.0324 | 0.0457 | 0.2471 | 1.0398(0.9894-1.1112) |
| Infections of breast associated with childbirth | Genus.Anaerotruncus | 8 | IVW | 0.0512 | 0.0287 | 0.2476 | 1.0578(0.9881-1.1128) |
| Infections of breast associated with childbirth | Genus.Butyricimonas. | 8 | IVW | 0.0187 | 0.0201 | 0.8769 | 1.0034(0.9687-1.0648) |
| Infections of breast associated with childbirth | Order.Coriobacteriales | 8 | IVW | 0.0762 | 0.0369 | 0.3776 | 1.0659(0.9776-1.1135) |
| Infections of breast associated with childbirth | Order.Pasteurellales | 8 | IVW | 0.0028 | 0.0472 | 0.7659 | 1.0043(0.9787-1.0673) |
| Infections of breast associated with childbirth | Order.Verrucomicrobiales | 8 | IVW | 0.0701 | 0.0546 | 0.1897 | 1.0739(0.9787-1.0600) |
