# Supplementary material for: Exploring the relationship between gut microbiota and breast diseases using Mendelian randomization analysis
Source: Front Med (Lausanne). 2024 Nov 26;11:1450298. doi: 10.3389/fmed.2024.1450298 (PMC11654425; doi:10.3389/fmed.2024.1450298)
Supplement: Supplementary file 3 [file Table_3.DOCX]

Supplemental Table 3. The statistical code of the Mendelian Randomization Analysis between Gut Microbiota and Breast Diseases.

#library(TwoSampleMR)

#library(ggplot2)

#setwd("X1") X1——refers to the location where the file is located；

#expo_rt<- read_exposure_data(

filename = "X2.txt",

sep = "\t",

snp_col = "rsID",

beta_col = "beta",

se_col = "SE",

effect_allele_col = "eff.allele",

other_allele_col = "re.allele",

pval_col = "P",

samplesize_col = "N"

) X2—— refers to the file name of the gut microbiota data downloaded from the GWAS database；

#expo_rt<- expo_rt[expo_rt$pval.exposure < 1e-5,]

#expo_rt <- clump_data(expo_rt,clump_kb = 10000,

clump_r2 = 0.001)

#write.table(expo_rt, "exposure.txt",row.names = F,sep = "\t",quote = F)

#outc_rt <- extract_outcome_data(

snps = expo_rt$SNP,

outcomes = "ieu-a-1126")

#harm_rt <- harmonise_data(

exposure_dat = expo_rt,

outcome_dat = outc_rt,action=1)

#write.table(harm_rt, "harmonise.txt",row.names = F,sep = "\t",quote = F)

#mr_result<- mr(harm_rt)

#View(mr_result)

#OR=generate_odds_ratios(mr_result)

#OR$pval=sort(OR$pval)

#OR$FDR=p.adjust(OR$pval,method = "fdr",n=119)

#write.table(OR[,5:ncol(OR)],"OR.txt",row.names = F,sep = "\t",quote = F)

#mr_heterogeneity(harm_rt)

#run_mr_presso(harm_rt,NbDistribution = 1000)

#mr_pleiotropy_test(harm_rt)

#singlesnp_res<- mr_singlesnp(harm_rt)

#View(singlesnp_res)

#singlesnpOR=generate_odds_ratios(singlesnp_res)

#write.table(singlesnpOR,"singlesnpOR.txt",row.names = F,sep = "\t",quote = F)

#sen_res<- mr_leaveoneout(harm_rt)

#View(sen_res)

#p1 <- mr_scatter_plot(my_mr_result, harm_rt)

#p1[[1]]

#ggsave(p1[[1]], file="scatterxinji9-19.pdf", width=8, height=8)

#p2 <- mr_forest_plot(singlesnp_res)

#p2[[1]]

#ggsave(p2[[1]], file="forest.pdf", width=8, height=8)

#p3 <- mr_leaveoneout_plot(sen_res)

#p3[[1]]

#ggsave(p3[[1]], file="sensitivity analysisxinji9-19.pdf", width=8, height=8)

#res_single <- mr_singlesnp(harm_rt)

#p4 <- mr_funnel_plot(singlesnp_res)

#p4[[1]]

#ggsave(p4[[1]], file="funnel plotxinji9-19.pdf", width=8, height=8)
