# Supplementary material for: Exploring the relationship between gut microbiota and breast diseases using Mendelian randomization analysis
Source: Front Med (Lausanne). 2024 Nov 26;11:1450298. doi: 10.3389/fmed.2024.1450298 (PMC11654425; doi:10.3389/fmed.2024.1450298)

Supplemental Figure S1. Scatter plot of the association between gut microbiota and overall breast cancer. (A) *Genus.Sellimonas*; (B) *Genus.Dorea*; (C) *Genus.Paraprevotella*; (D) *Family.Rikenellaceae;* (E) *Family.Ruminococcaceae;* (F) *Family.Streptococcaceae;* (G) *Phylum.Bacteroidetes*

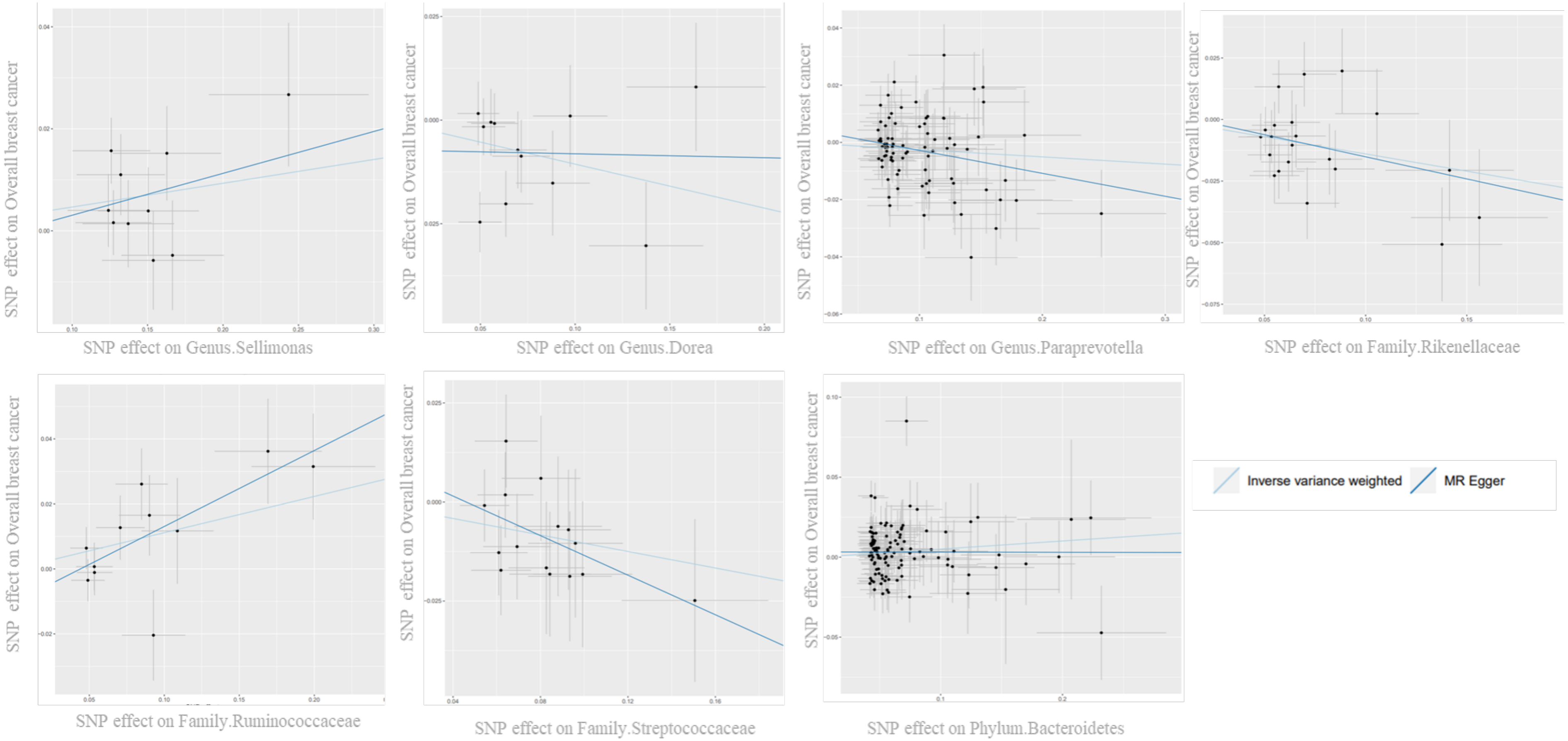

Supplemental Figure S2. Scatter plot of the association between gut microbiota and ER(+)、ER(-) breast cancer. (A) *Genus.Sellimonas*; (B)

Genus.Adlercreutzia; (C) *Genus.CandidatusSoleaferrea*; (D) *Genus.Paraprevotella;* (E) *Family.Rikenellaceae;* (F) *Order.Bifidobacteriales;* (G) *Genus.Dorea*

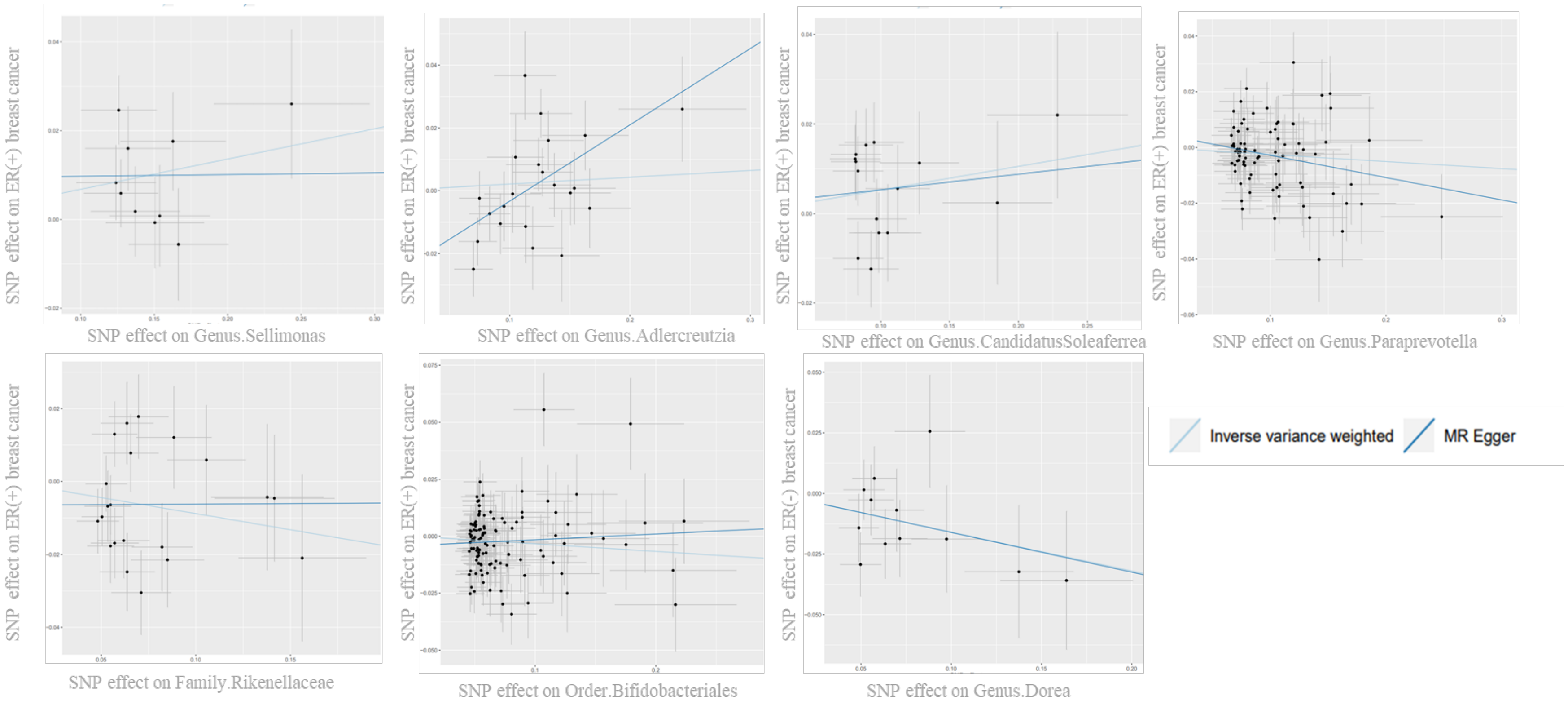

Supplemental Figure S3. Scatter plot of the association between gut microbiota and breast cyst and inflammatory disorders of breast. (A) *Genus.Eubacteriumruminantiumgroup*; (B) *Genus.Lactococcus*; (C) *Family.Alcaligenaceae.*

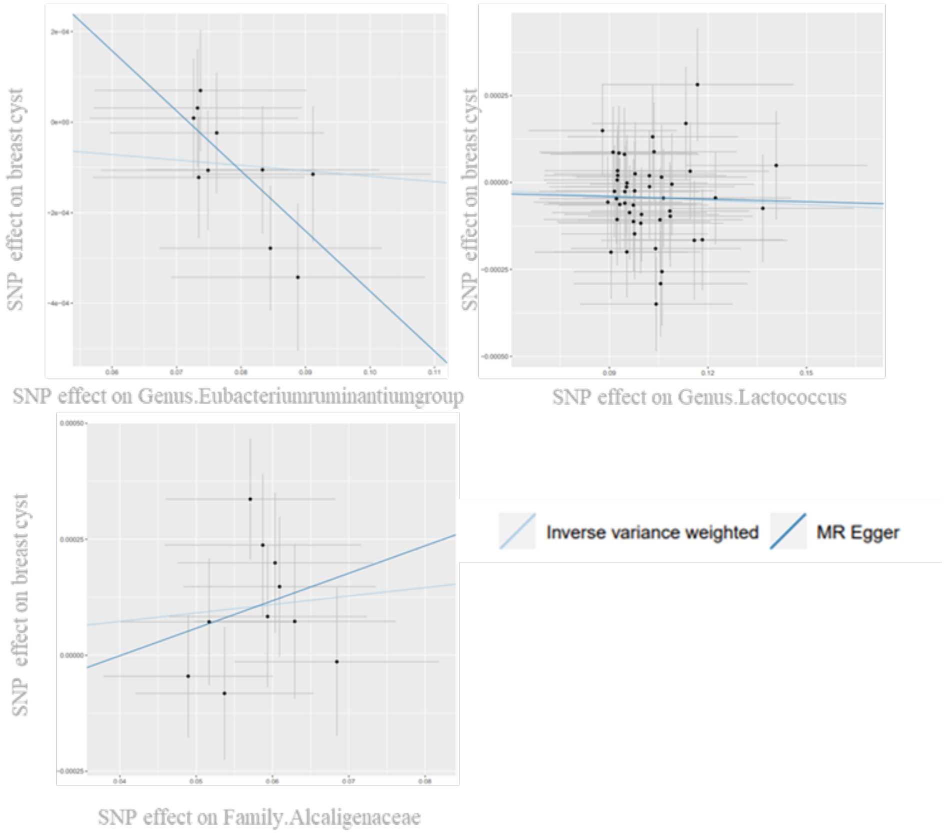

Supplemental Figure S4. Scatter plot of the association between gut microbiota and inflammatory disorders of breast. (A) *Family.Prevotellaceae.*

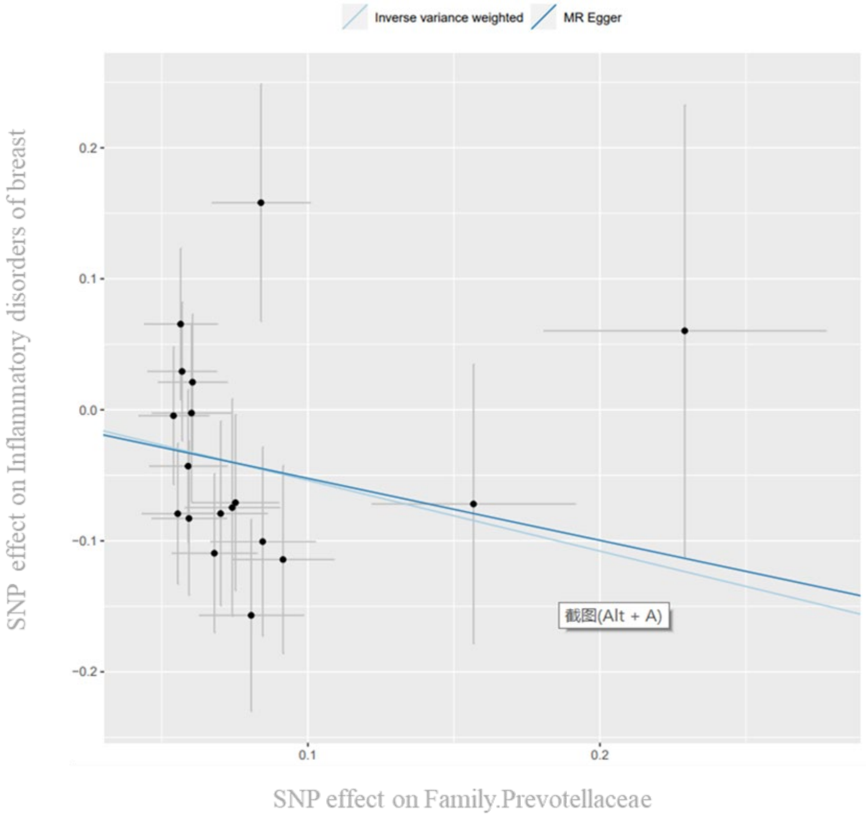

Supplemental Figure S5. Scatter plot of the association between gut microbiota and Infections of breast associated with childbirth. (A) *Genus.Anaerofilum*; (B) *Genus.Anaerotruncus*; (C) *Genus.Butyricimonas*; (D) *Order.Coriobacteriales;* (E) *Order.Pasteurellales;* (F) *Order.Verrucomicrobiales.*

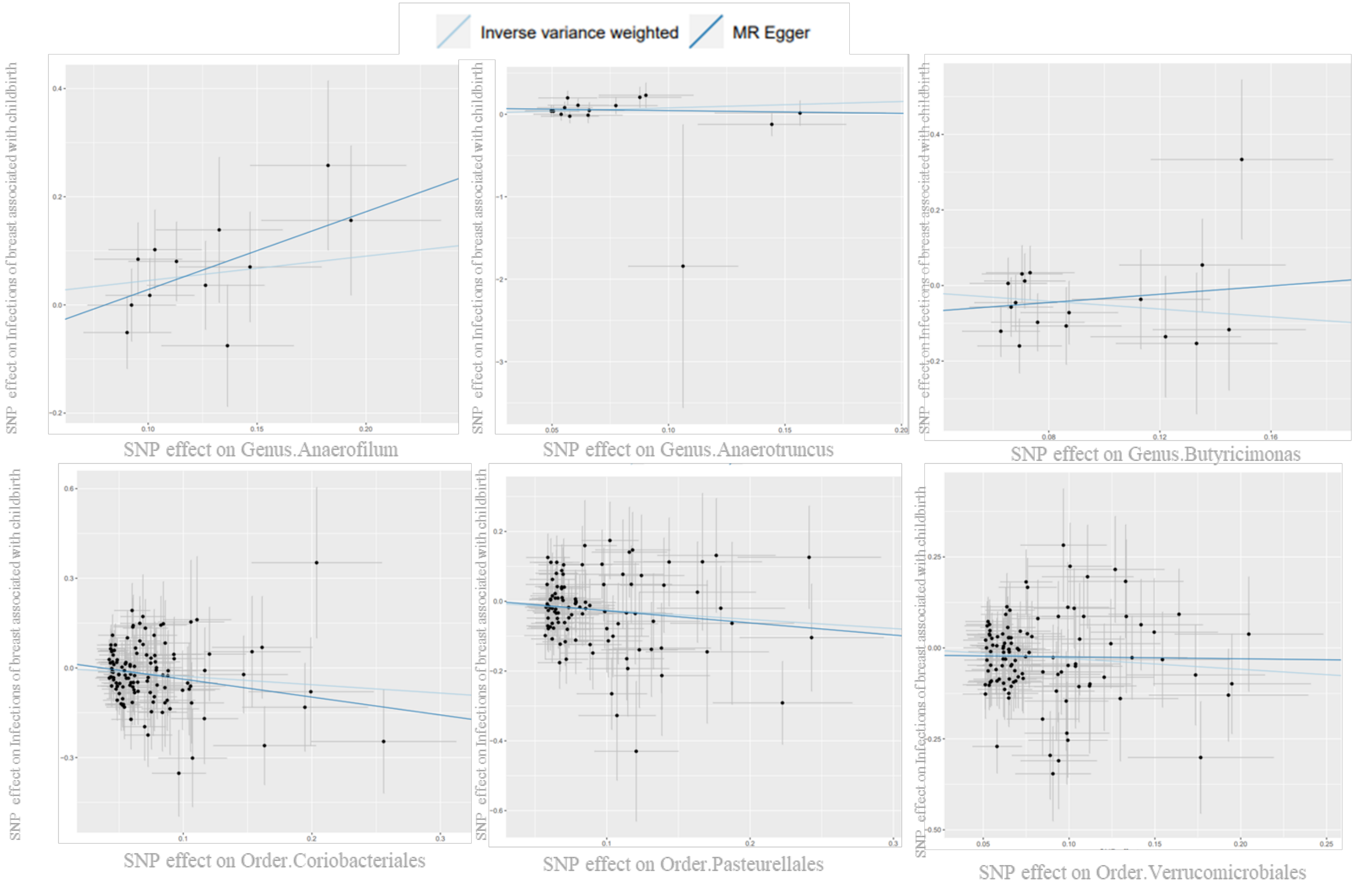

Supplemental Figure S6. Leave-one-out sensitivity analysis for the association between gut microbiota and overall breast cancer. (A) *Genus.Sellimonas*; (B) *Genus.Dorea*; (C) *Genus.Paraprevotella*; (D) *Family.Rikenellaceae;* (E) *Family.Ruminococcaceae;* (F)

Family.Streptococcaceae; (G) *Phylum.Bacteroidetes*

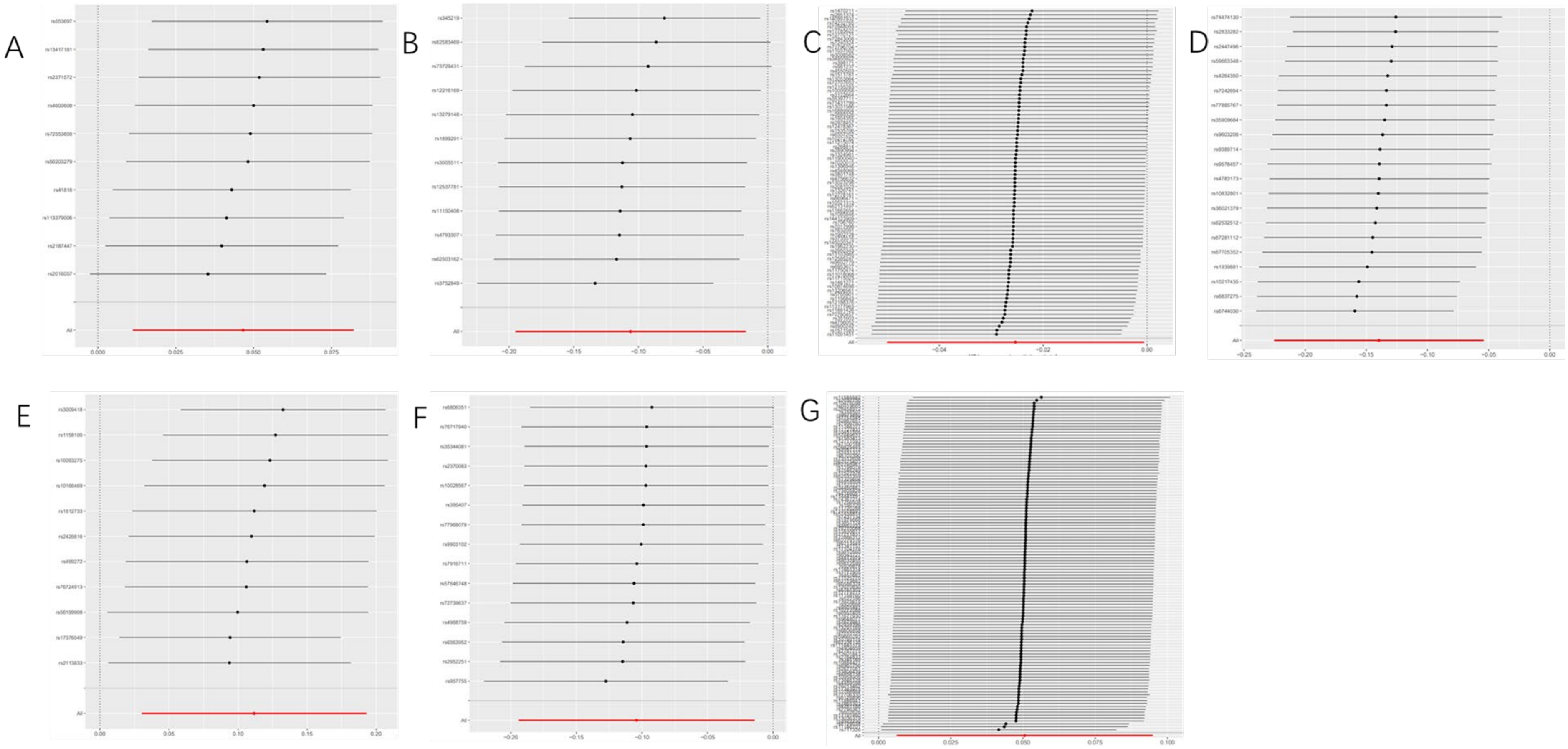

Supplemental Figure S7. Leave-one-out sensitivity analysis for the association between gut microbiota and ER(+)、ER(-) breast cancer. (A)

Genus.Sellimonas; (B) *Genus.Adlercreutzia*; (C) *Genus.CandidatusSoleaferrea*; (D) *Genus.Paraprevotella;* (E) *Family.Rikenellaceae;* (F) *Order.Bifidobacteriales;* (G) *Genus.Dorea*

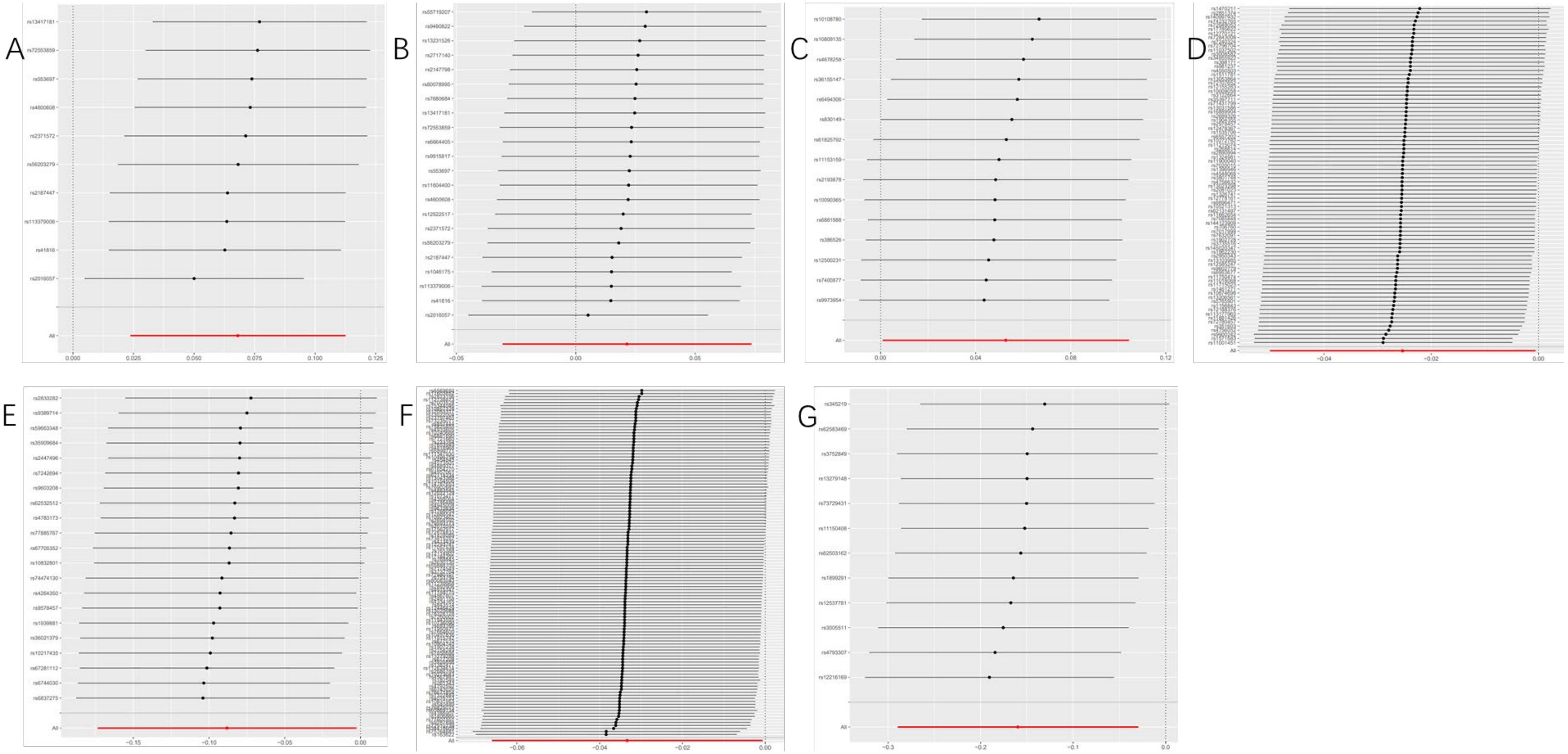

Supplemental Figure S8. Leave-one-out sensitivity analysis for the association between gut microbiota and breast cyst and inflammatory disorders of breast. (A) *Genus.Eubacteriumruminantiumgroup*; (B) *Genus.Lactococcus*; (C) *Family.Alcaligenaceae.*

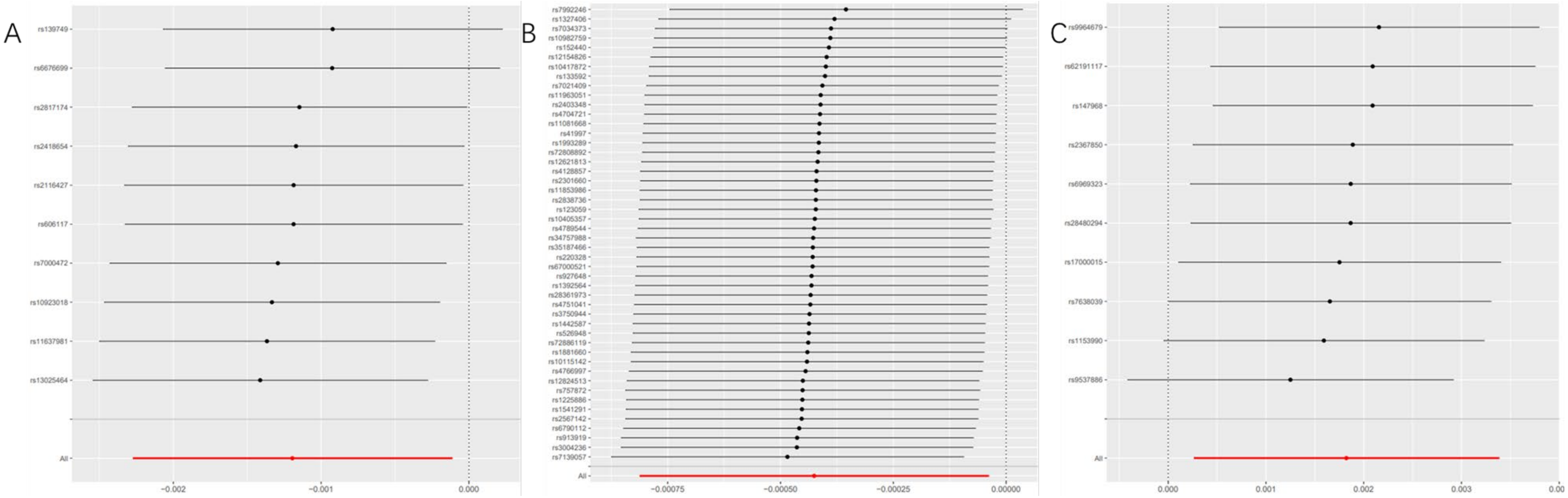

Supplemental Figure S9. Leave-one-out sensitivity analysis for the association between gut microbiota and inflammatory disorders of breast. (A) *Family.Prevotellaceae.*

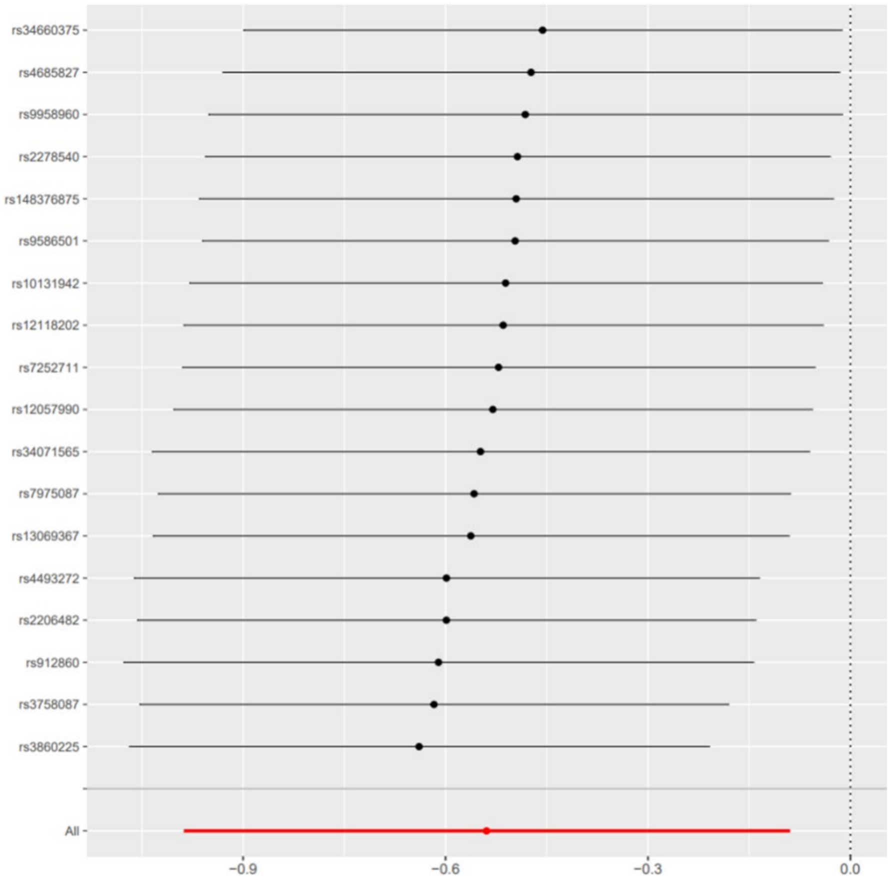

Supplemental Figure S10. Leave-one-out sensitivity analysis for the association between gut microbiota and Infections of breast associated with

childbirth. (A) *Genus.Anaerofilum*; (B) *Genus.Anaerotruncus*; (C) *Genus.Butyricimonas*; (D) *Order.Coriobacteriales;* (E) *Order.Pasteurellales;* (F) *Order.Verrucomicrobiales*

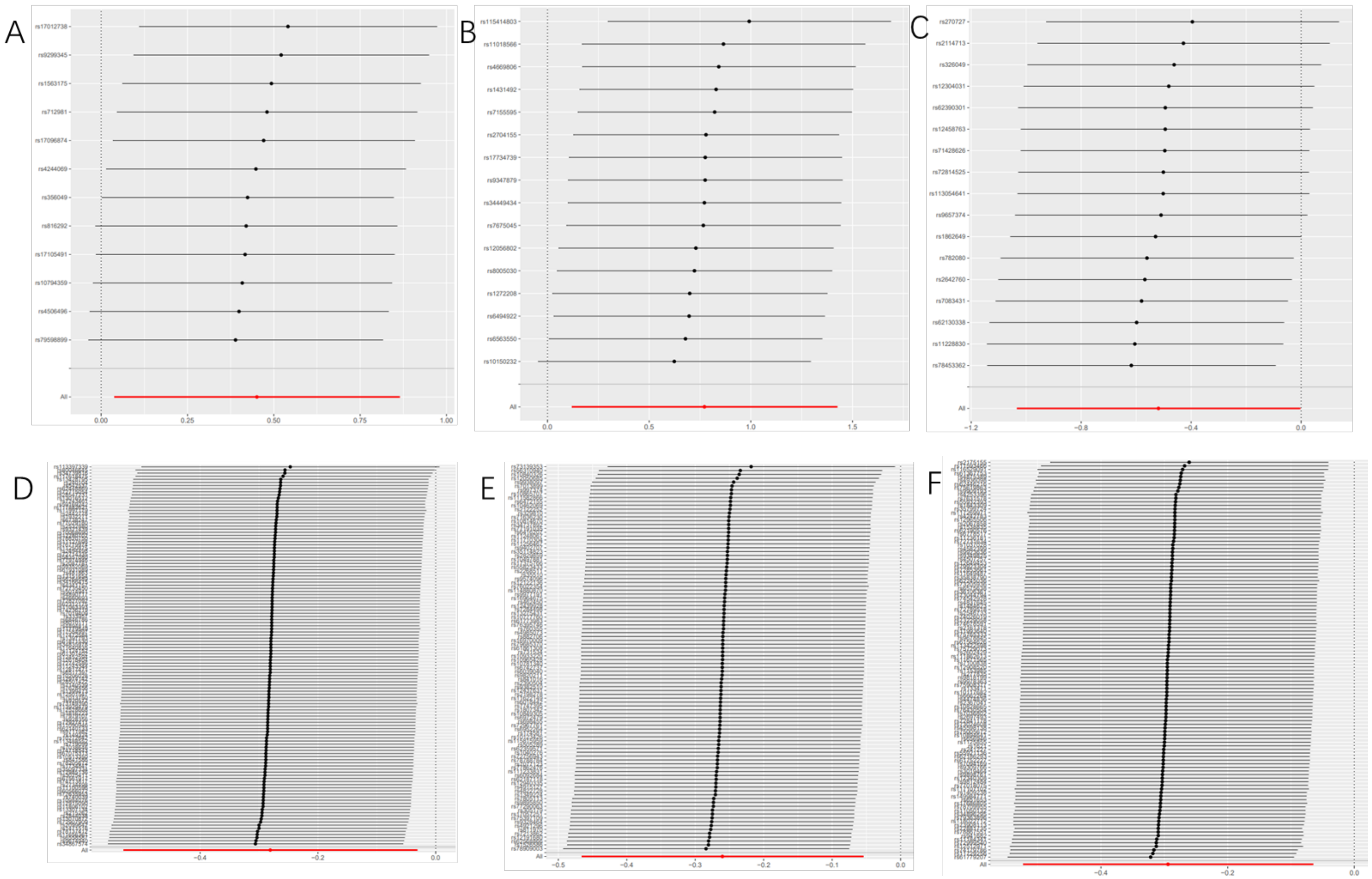

Supplement: Supplementary file 5 [file Data_Sheet_2.PDF]
